# Supplementary material for: Genetic architecture of a key reproductive isolation trait differs between sympatric and non-sympatric sister species of Lake Victoria cichlids
Source: Proc Biol Sci. 2020 Apr 8;287(1924):20200270. doi: 10.1098/rspb.2020.0270 (PMC7209058; doi:10.1098/rspb.2020.0270)
Supplement: Appendix, Figure S1, Supplementary Tables 1-5 [file rspb20200270supp1.pdf]

Electronic supplementary material

Genetic architecture of a key reproductive isolation trait differs between sympatric and non-sympatric sister species of Lake Victoria cichlids

DOI: 10.1098/rspb.2020.0270

Appendix S1

We normalized DNA concentration for all samples in one library prior to restriction enzyme digestion. 4-48 individuals carrying TruSeq P1-adaptors and different custom 5bp-8bp barcodes were pooled into one library (see Table S5). For shearing we used a Covaris M220 Focused-ultrasonicator. Sheared fragments between 300-700bp were selected on a SageELF machine. After ligation of the TruSeq P2-adaptors each library was amplified in four aliquots of 50 µl.

Appendix S2

In a first step, PhiX reads were removed using Bowtie2 v2.3.2 [1]. Reads were then demultiplexed and trimmed to 85 bp with process_radtags implemented in stacks v.1.40 [2]. The FASTQ quality filter (http://hannonlab.cshl.edu/ fastx_toolkit/index.html) was used to filter reads for a minimum quality of 10 at all bases and of 30 in at least 95% of the reads.

After alignment to the *Pundamilia nyererei* reference genome [3] with Bowtie2 v2.3.2 [1], base quality recalibration was performed using the BaseRecalibrator and PrintReads modules of the Genome Analysis Toolkit v3.7 [4].

Genotyping (only for uniquely aligned reads) was done with (GATK Unified Genotyper v3.7. [4]). In the red-dorsum x blue cross, this included the 231 focal individuals (225 F2s belonging to two families, of which 44 females, the four F1 parents and the two F0 grandparents), plus 8 individuals (seven F2s and one F1 parent) belonging to a smaller family with the same grandparents and one shared F1 parent, as well as 27 individuals (22 F2s and five F1 parents) with different grandparents of the same two species. The 35 additionally genotyped individuals with different parents/grandparents were not used in the following steps or any analyses. In the red-chest x blue cross, genotyping included a total of 211 individuals (205 F2 individuals belonging to two families, of which 68 females, the four F1 parents, and the two F0 parents).

The vcf file filtering procedure using Bcftools implemented in Samtools v.1.8 [5] and Vcftools v.0.1.14 [6] was the following: after filtering out sites with >50% missing data we removed individuals with a mean depth of <12 and / or >50% missing data. We removed sites within 10 bp of indels and kept only bi-allelic SNPs with a mean genotype depth of less than 1.5 times the interquartile range from the mean (as sites with a greater depth are expected to be enriched for duplicates), and set genotypes with a depth of <10 to missing. Individuals were then checked for PCR duplicates and removed if the heterozygous read balance was heavily skewed. Sites were then again filtered for missing data of max. 50% and for a minor allele frequency of min. 0.05.

For the red-dorsum x blue cross, this resulted in 10,598 SNPs over the remaining 224 individuals (218 F2s, of which 43 females, four F1 parents, and two F0 grandparents). As only 566 of these SNPs appeared to have alternative homozygous genotypes between the two F0 grandparents, we additionally applied a custom allelic balance filter on these two individuals, where heterozygous genotypes failing a binomial test on the genotype distributions were set to homozygous. After this correction, 1,326 SNPs appeared as homozygous fixed in the F0, which is closer to our expectations based on other recent QTL mapping studies on cichlids using RAD sequencing data e.g. [3,7]. 368 of these SNPs were heterozygous as expected in all four F1 parents and were used in linkage map construction.

For the red-chest x blue cross, this resulted in 9,990 SNPs over the remaining 190 individuals (186 F2s, of which 57 females, two F1 parents of one family (the two of the other family were removed in the filtering procedures due to low quality) and two F0 grandparents). 3,473 SNPs were retained as alternative homozygous between the two F0 grandparents after applying the same allelic balance filter as for the red-dorsum x blue cross. 2,358 of these were heterozygous as expected in both F1s and used in linkage map construction.

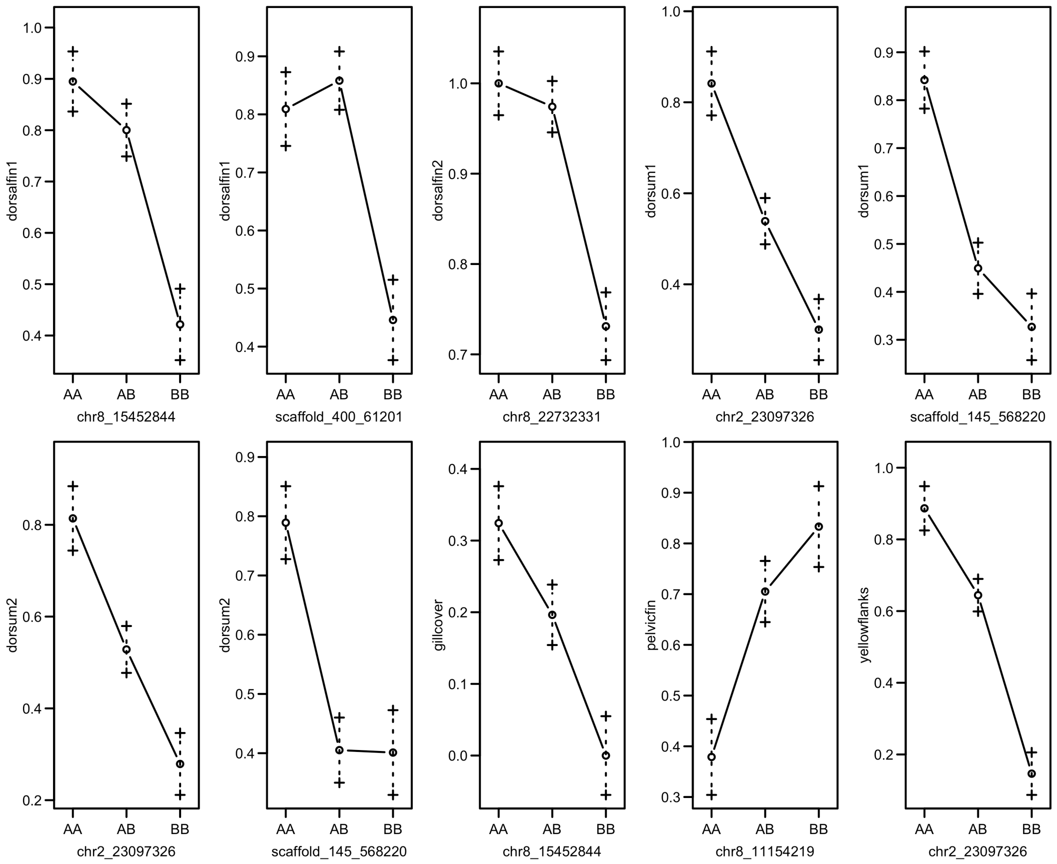

Figure S1. Effect plots at the markers on the QTL peaks for all significant QTLs in the red-dorsum x blue cross. The genotype AA corresponds to homozygous as *P.* sp. "pundamilia-like", BB to homozygous as *P*. sp. "nyererei-like".

Table S1. Locations and effects of QTLs for the presence/absence of red colour on different body parts, for yellow flanks, and for a phenotypic hybrid index.

trait	# F2s	marker (nearest marker)	Pun-LG	Ore-LG	cM	95% CI (cM)	nearest markers	LOD	P -value	PVE
red-dorsum x blue cross										
dorsal fin 1	140	c8.loc38 (chr8_15452844)	8	19	38.0	35-48.3	chr8_14022265 - scaffold_261_382784	5.64	<0.001	16.77
dorsal fin 1	140	scaffold_400_61201	10	23	12.7	2-25	scaffold_844_38876 - chr10_30070154	5.16	0.001	15.51
dorsal fin 2	172	c8.loc44 (chr8_22732331)	8	19	44.0	35-48.3	chr8_14022265 - scaffold_261_382784	6.44	<0.001	15.29
dorsum 1	171	chr2_23097326	2	7	13.2	0-17	chr2_14394351 - chr2_34782227	5.93	0.001	14.82
dorsum 1	171	scaffold_145_568220	6	11	5.4	0-8	chr6_2497279 - chr6_4731967	7.07	<0.001	17.35
dorsum 2	171	chr2_23097326	2	7	13.2	0-16	chr2_14394351 - chr2_23097326	5.73	0.002	14.36
dorsum 2	171	scaffold_145_568220	6	11	5.4	0-8	chr6_249727 - chr6_4731967	4.92	0.005	12.43
gill cover	174	c8.loc38 (chr8_15452844)	8	19	38.0	15-48.3	chr8_8525018 - scaffold_261_382784	5.08	0.002	12.57
pelvic fin	126	c8.loc30 (chr8_11154219)	8	19	30.0	24-48.3	chr8_8524751 - scaffold_261_382784	4.22	0.015	14.27
pelvic fin	126	c12.loc52 (chr12_33255157)	12	17	52	30-58	chr12_25602998 - chr12_33255157	3.18	0.088	10.75
yellow flanks	174	c2.loc12 (chr2_23097326)	2	7	12.0	8-16	chr2_17806364 - chr2_23097326	13.27	<0.001	29.49
hybrid index	119	c2.loc12 (chr2_23097326)	2	7	12	0-15	chr2_14394351 - chr2_23097326	7.14	<0.001	17.20
hybrid index	119	chr6_2497279	6	11	0	0-8	chr6_2497279 - chr6_4731967	3.35	0.084	8.50
hybrid index	119	scaffold_261_382784	8	19	48.3	33-48.3	chr8_13110496 -scaffold_261_382784	4.41	0.01	10.97
hybrid index	119	c18.loc9 (chr18_9611117)	18	6	9	3-14	chr18_2214232- chr18_9611117	5.43	0.003	13.28
pelvic fin (add/int)	126	chr8_19963137	8	19	40.4			5.06	0.022	13.76
pelvic fin (add/int)	126	chr22_6708694	22	12	24.7			4.33	0.079	6.71
red-chest x blue cross										
throat (add)	125	chr7_9610352	7	15	9.9			3.77	0.064	8.31
head *	123	c9.loc21 (chr9_11198659)	9	18	21			3.50	0.057	9.85

F2s, number of F2 males with data; (nearest marker), nearest RAD marker to a QTL at an interpolated marker (where genotypes were inferred by maximum likelihood estimation under a mixture model, using hidden Markov models); Pun-LG, linkage group number corresponding to the anchored *Pundamilia nyererei* reference genome [3]; Ore-LG, linkage group number corresponding to the *Oreochromis niloticus* reference genome [8]; cM, position in centiMorgan; 95% CI , 95% approximate Bayesian credible interval (in cM); PVE, percent variance explained; add, accounting for family as additive covariate; int, accounting for family as additive and as interactive covariate; * found in 2/5 mappings with subsampled map (see also Table S4)

Table S2. Results from two-dimensional two-QTL scans

	trait	chr 1	chr 2	pos 1f	pos 2f	lod full	pval full	lod fv1	pval fv1	lod int	pval int	pos 1a	pos 2a	lod add	pval add	lod av1	pval av1
red-dorsum x blue cross	dorsal fin 1	8	10	48	12	10.86	0.000	5.32	0.576	1.80	1.000	48	12	9.06	0.000	3.52	0.017
	dorsal fin 1	8	12	39	33	11.85	0.000	6.30	0.079	3.87	0.982	39	36	7.98	0.000	2.43	0.347
	dorsal fin 2	1	8	21	42	9.36	0.000	3.15	1.000	-0.04	1.000	18	42	9.40	0.000	3.19	0.036
	dorsum 1	2	6	12	6	14.93	0.000	7.90	0.007	0.81	1.000	0	6	14.12	0.000	7.09	0.000
	dorsum 1	2	11	12	3	10.29	0.004	4.45	0.994	1.31	1.000	12	3	8.97	0.000	3.14	0.045
	dorsum 1	2	18	12	6	9.13	0.028	3.30	1.000	0.20	1.000	12	6	8.93	0.000	3.10	0.050
	dorsum 1	6	18	6	9	12.07	0.000	5.04	0.847	0.81	1.000	6	9	11.26	0.000	4.23	0.003
	dorsum 2	2	6	12	0	12.48	0.000	6.86	0.066	1.36	1.000	0	0	11.12	0.000	5.50	0.000
	dorsum 2	6	18	6	9	8.81	0.041	3.90	1.000	0.94	1.000	6	9	7.87	0.003	2.96	0.083
	gill cover	6	8	0	42	9.15	0.007	4.09	1.000	0.29	1.000	0	42	8.85	0.000	3.80	0.008
	pelvic fin	4	8	24	21	10.81	0.000	6.59	0.086	4.87	0.556	39	39	5.94	0.055	1.71	0.945
	pelvic fin	8	12	30	36	10.32	0.001	6.10	0.216	1.85	1.000	30	36	8.47	0.001	4.25	0.000
	yellow flanks	2	18	12	15	17.93	0.000	4.66	0.976	0.70	1.000	12	15	17.24	0.000	3.96	0.003
red-chest x blue cross																	
	cheek	1	4	15	24	8.78	0.092	7.77	0.016	7.23	0.011	9	27	1.55	1	0.53	1
	throat	1	4	15	30	9.11	0.067	8.03	0.012	7.70	0.006	30	54	1.41	1	0.33	1
	yellow flanks	3	11	54	51	9.37	0.033	7.01	0.088	6.04	0.127	54	60	3.32	0.992	0.97	1

lod.full, comparing the full model (two QTLs with interaction) to the null model (no QTLs); lod.fv1, comparing the full model to the best single QTL model for a respective trait; lod.int, comparing the full model to the additive (two QTLs with no interaction) model i.e. indicating evidence for epistasis; lod.add, comparing the additive model to the null model; lod.av1 comparing the additive model to the best single QTL model.

Table S3. QTL mapping in the red-dorsum x blue cross repeated five times with randomly sampled 125 F2 males. Only traits with scores below a genome-wide significance threshold of 0.1 are shown.

	chr	pos	dorsalfin1	pval	dorsalfin2	pval	dorsum1	pval	dorsum2	pval	gillcover	pval	pelvicfin	pval	yellowflanks	pval
1																
c2.loc1	2	1.0	1.15	1.000	0.92	1.000	4.82	0.007	4.28	0.012	0.63	1.000	0.10	1.000	9.54	0.000
chr2_23097326	2	13.2	0.05	1.000	0.01	1.000	4.20	0.018	4.13	0.018	0.94	1.000	0.16	1.000	11.38	0.000
scaffold_145_568220	6	5.4	0.35	1.000	0.42	1.000	4.43	0.012	3.62	0.042	2.83	0.230	0.77	1.000	0.35	1.000
chr6_4731967	6	8.5	0.07	1.000	0.08	1.000	4.29	0.014	3.88	0.029	2.24	0.604	0.65	1.000	0.81	1.000
chr8_8525018	8	21.0	1.83	0.852	1.14	0.999	1.56	0.953	1.63	0.932	4.69	0.005	3.78	0.039	0.14	1.000
chr8_11154219	8	29.4	2.17	0.656	2.32	0.493	0.92	1.000	0.81	1.000	3.22	0.117	5.16	0.001	0.22	1.000
chr8_19857096	8	42.6	4.04	0.018	3.47	0.022	1.11	1.000	1.15	1.000	4.04	0.023	4.09	0.021	1.11	1.000
chr16_8832104	16	0.0	0.34	1.000	0.62	1.000	2.89	0.193	2.68	0.266	3.33	0.099	1.73	0.887	1.25	0.994
chr18_579662	18	0.0	1.17	1.000	1.23	0.999	3.28	0.091	1.66	0.913	1.07	1.000	1.44	0.977	2.09	0.629
2																
c2.loc12	2	12.0	0.04	1.000	0.22	1.000	4.22	0.015	4.63	0.002	0.96	1.000	1.16	1.000	9.73	0.000
chr2_23097326	2	13.2	0.05	1.000	0.22	1.000	4.31	0.012	4.73	0.000	0.98	1.000	1.15	1.000	9.63	0.000
scaffold_145_568220	6	5.4	0.03	1.000	0.41	1.000	6.42	0.000	4.76	0.000	2.70	0.303	0.38	1.000	0.51	1.000
c8.loc42	8	42.0	2.57	0.364	2.96	0.074	0.28	1.000	0.24	1.000	4.40	0.011	2.36	0.490	1.70	0.896
chr8_19857096	8	42.6	2.67	0.301	3.10	0.053	0.29	1.000	0.25	1.000	4.39	0.011	2.35	0.490	1.76	0.865
3																
c2.loc12	2	12.0	0.04	1.000	0.22	1.000	4.22	0.015	4.63	0.002	0.96	1.000	1.16	1.000	9.73	0.000
chr2_23097326	2	13.2	0.05	1.000	0.22	1.000	4.31	0.012	4.73	0.000	0.98	1.000	1.15	1.000	9.63	0.000

scaffold_145_568220	6	5.4	0.03	1.000	0.41	1.000	6.42	0.000	4.76	0.000	2.70	0.303	0.38	1.000	0.51	1.000
c8.loc42	8	42.0	2.57	0.364	2.96	0.074	0.28	1.000	0.24	1.000	4.40	0.011	2.36	0.490	1.70	0.896
chr8_19857096	8	42.6	2.67	0.301	3.10	0.053	0.29	1.000	0.25	1.000	4.39	0.011	2.35	0.490	1.76	0.865
4																
c2.loc11	2	11.0	0.36	1.000	0.55	1.000	5.03	0.002	4.41	0.011	1.30	0.999	0.02	1.000	9.85	0.000
c2.loc13	2	13.0	0.33	1.000	0.37	1.000	5.06	0.001	4.46	0.011	1.21	1.000	0.01	1.000	9.61	0.000
scaffold_145_568220	6	5.4	0.23	1.000	0.28	1.000	5.77	0.000	3.40	0.076	1.41	0.991	0.01	1.000	0.14	1.000
chr8_11154219	8	29.4	1.73	0.908	2.67	0.257	0.39	1.000	0.31	1.000	2.03	0.742	4.23	0.015	0.28	1.000
chr8_22732331	8	45.1	3.10	0.138	4.04	0.003	0.12	1.000	0.33	1.000	3.42	0.060	3.55	0.047	1.83	0.843
scaffold_261_382784	8	48.3	3.11	0.135	3.97	0.005	0.26	1.000	0.56	1.000	3.55	0.045	3.75	0.033	1.31	0.993
c10.loc13	10	13.0	5.31	0.000	2.91	0.135	0.21	1.000	0.13	1.000	0.13	1.000	1.04	1.000	0.12	1.000
c12.loc53	12	53.0	0.39	1.000	0.07	1.000	0.32	1.000	0.50	1.000	0.47	1.000	*4.99*	*0.000*	1.54	0.960
5																
c2.loc10	2	10.0	0.76	1.000	0.33	1.000	5.62	0.002	6.10	0.002	0.92	1.000	0.03	1.000	9.21	0.000
chr2_23097326	2	13.2	0.76	1.000	0.45	1.000	5.94	0.002	6.44	0.001	1.02	1.000	0.02	1.000	8.91	0.000
scaffold_145_568220	6	5.4	0.09	1.000	0.03	1.000	5.41	0.002	3.52	0.053	1.67	0.928	0.12	1.000	0.05	1.000
c6.loc7	6	7.0	0.06	1.000	0.10	1.000	5.45	0.002	3.51	0.056	1.54	0.963	0.08	1.000	0.16	1.000
c8.loc38	8	38.0	3.95	0.023	3.96	0.006	0.10	1.000	0.01	1.000	3.16	0.151	2.80	0.234	0.17	1.000
c8.loc43	8	43.0	3.82	0.033	4.28	0.005	0.13	1.000	0.01	1.000	2.97	0.205	2.96	0.170	1.07	1.000
chr8_22732331	8	45.1	3.87	0.028	3.76	0.016	0.10	1.000	0.00	1.000	3.18	0.148	3.32	0.089	0.89	1.000
scaffold_261_382784	8	48.3	3.63	0.047	3.45	0.033	0.11	1.000	0.03	1.000	3.46	0.080	2.90	0.193	0.66	1.000

Colour background indicates (marginally) significant LOD scores/p-values. Green: QTLs known from scanone results (darkgreen are the known significant QTLs that were recovered as significant QTLs (i.e. pos within the 95% CI of the known significant QTL)); Blue: QTLs known from scantwo results; Red: not seen before.

Table S4. QTL mapping in the red-chest x blue cross repeated five times with a randomly subsampled linkage map to match the number markers of the red-dorsum x blue cross. Only traits with LOD scores below a genome-wide significance threshold of 0.1 are shown.

locus	chr	pos	head	pval
1				
There were no LOD peaks above the threshold.				
2				
There were no LOD peaks above the threshold.				
3				
c9.loc21 (chr9_11198659)	9	21	3.50	0.057
4				
c9.loc20 (chr9_9921245)	9	20	3.23	0.089
**5**				
There were no LOD peaks above the threshold.				

Table S5. Overview of RAD sequencing libraries with number of individuals.

library	# total	# F0	# F1	# F2	platform	read length
red-chest x blue cross, see also [3]						
GQI21	47	2	2	43	Bern	100
GQI22	30	2	-	28	Bern	100
GQI26	10	-	-	10	Bern	100
GQI28	40	-	-	40	Bern	100
GQI29	4	4	-	-	Bern	100
GQI66	48*	-	-	36	Bern	100
GQI67	47		-	47	Bern	100
GQI99	37	-	-	37	Lausanne	100
GQI100b	37	-	-	37	Lausanne	100
red-dorsum x blue cross						
GQI101	8	2	6 (2 red-chest x blue)	-	Lausanne	100
GQI102	48	-	-	48	Lausanne	100
GQI123	47	-	-	47	Lausanne	100
GQI124	47	-	3	44	Lausanne	100
GQI134	47	-	-	47	Lausanne	100
GQI140	47	-	2	45	Lausanne	100
GQI144	47	-	1	46	Lausanne	125
GQI146	27**	-	-	15	Lausanne	125

numbers include individuals that were resequenced if they had low quality or low read number in the first run; *contains 12 individuals of a different species; **contains 12 F0/F1 individuals of a different cross

References for Appendix / Supplementary Materials

1. Langmead B, Salzberg SL. 2012 Fast gapped-read alignment with Bowtie 2. *Nat. Methods* **9**, 357–359. (doi:10.1038/nmeth.1923)
2. Catchen J, Hohenlohe PA, Bassham S, Amores A, Cresko WA. 2013 Stacks: an analysis tool set for population genomics. *Mol. Ecol.* **22**, 3124–3140. (doi:10.1111/mec.12354)
3. Feulner PGD, Schwarzer J, Haesler MP, Meier JI, Seehausen O. 2018 A Dense Linkage Map of Lake Victoria Cichlids Improved the *Pundamilia* Genome Assembly and Revealed a Major QTL for Sex-Determination. *G3; Genes|Genomes|Genetics* **8**, 2411–2420. (doi:10.1534/g3.118.200207)
4. McKenna A *et al.* 2010 The Genome Analysis Toolkit: A MapReduce framework for analyzing next-generation DNA sequencing data. *Genome Res.* **20**, 1297–1303. (doi:10.1101/gr.107524.110)
5. Li H, Handsaker B, Wysoker A, Fennell T, Ruan J, Homer N, Marth G, Abecasis G, Durbin R. 2009 The Sequence Alignment/Map format and SAMtools. *Bioinformatics* **25**, 2078–2079. (doi:10.1093/bioinformatics/btp352)
6. Danecek P *et al.* 2011 The variant call format and VCFtools. *Bioinformatics* **27**, 2156–2158. (doi:10.1093/bioinformatics/btr330)
7. Henning F, Machado-Schiaffino G, Baumgarten L, Meyer A. 2017 Genetic dissection of adaptive form and function in rapidly speciating cichlid fishes. *Evolution* **71**, 1297–1312. (doi:10.1111/evo.13206)
8. Brawand D *et al.* 2014 The genomic substrate for adaptive radiation in African cichlid fish. *Nature* **513**, 375–381. (doi:10.1038/nature13726)
